# Euthymic despite pain: the role of cognitive reappraisal and experiential avoidance in autoimmune inflammatory rheumatic diseases—a cross-sectional study

**DOI:** 10.3389/fpsyg.2024.1467555

**Published:** 2024-10-04

**Authors:** Francesco De Vincenzo, Luca Iani, Chiara Alessio, Luca Navarini, Damiano Currado, Annalisa Marino, Anna Contardi

**Affiliations:** ^1^Department of Human Sciences, European University of Rome, Rome, Italy; ^2^Rheumatology and Clinical Immunology, Department of Medicine, University of Rome "Campus Bio-Medico" School of Medicine, Rome, Italy; ^3^Clinical and Research Section of Rheumatology and Clinical Immunology, Fondazione Policlinico Universitario Campus Bio-Medico, Rome, Italy

**Keywords:** euthymia, chronic pain, arthritis, psychological flexibility, experiential avoidance, cognitive reappraisal, emotion regulation, moderated mediation

## Abstract

Pain is a central feature of inflammatory rheumatic diseases and is associated with psychological distress. Pain is widely recognized not as a mere physical sensation, but as a complex, multidimensional phenomenon with an affective component. A plethora of research has conceptualized adaptation to pain by focusing on minimizing the pain experience. However, pain in autoimmune inflammatory rheumatic diseases is often neither avoidable nor curable. This cross-sectional study aimed to investigate the processes explaining how pain intensity may be associated with low well-being and why some patients may live well despite pain. Drawing upon the psychological (in)flexibility model and the process model of emotion regulation, we propose that cognitive reappraisal moderates the association between pain and euthymia through experiential avoidance. Ninety-seven patients with rheumatoid arthritis, psoriatic arthritis, or axial spondyloarthritis were included for analyses (mean age = 53.4; mean time since diagnosis = 9.2 years). Most patients were women (75%), married/cohabitant (71%), and attended high school (47%). Results indicate that experiential avoidance may explain how severe pain is associated with lowered euthymia. This indirect negative effect of pain intensity on euthymia became non-significant at high levels of cognitive reappraisal, suggesting that cognitive reappraisal may serve as a protective factor for patients with autoimmune inflammatory rheumatic diseases. This study paves the way for future research in this promising context by providing an initial step towards integrating emotion regulation and psychological inflexibility in pain conditions.

## 1 Introduction

Research on chronic pain has mostly considered pain as a clinically relevant outcome (e.g., Martinez-Calderon et al., 2018a,b; Reiner et al., 2013). The identification of factors contributing to the experience of pain is crucial for developing targeted interventions and to identify vulnerable patients. However, research on characteristics that allow patients to live well despite pain is still limited (e.g., Flink et al., 2015; Peters et al., 2017). Studies addressing this issue are in line with the clinicians’ goal to improve patients’ quality of life, rather than merely striving to reduce chronic pain (Sullivan and Ballantyne, 2016). This is particularly relevant given that a significant number of patients suffering from autoimmune inflammatory rheumatic diseases—a family of autoimmune conditions primarily affecting the connective tissues and musculoskeletal organs—exhibit persistent residual pain despite the achievement of remission or low disease activity (Ishida et al., 2018; Liu et al., 2021). Some studies have demonstrated that advances in biological treatments for rheumatoid arthritis are still not enough to improve some relevant patient-reported outcomes. A recent meta-analysis investigated the temporal improvement of patient-reported outcomes over the last 30 years in light of new treatment strategies employed in early 2000s (Carpenter et al., 2020). It was found that adopting new therapy approaches [i.e., Treat-2-Target, and biologic Disease Modifying Anti-Rheumatic Drugs (DMARDs)] coincided with improvements in disease activity and physical function, but not in pain, functional disability, and mental well-being (Carpenter et al., 2020).

Pain is not merely a physical symptom (i.e., nociception); it also reflects unpleasant emotional states (i.e., affective-motivational dimension), and appraisals of meanings and consequences of pain (i.e., cognitive-evaluative dimension; Lumley et al., 2011; Melzack and Casey, 1968). The Dynamic Model of Effective Pain Adaptation emphasizes how persons adaptively respond to the pain experience, rather than focusing on pain itself (Sturgeon and Zautra, 2016). Dimensions of resilient pain adaptation include recovery, referring to the ways a person effectively returns to baseline levels of emotional and physical functioning (e.g., low pain intensity), and sustainability, reflecting the positive and meaningful engagement of a person despite the presence of pain (Sturgeon and Zautra, 2016). The latter may be operationalized as “the continuing experience of optimal emotional, psychological, and social well-being in the presence of pain” (Goubert and Trompetter, 2017, p. 3), that is flourishing (Keyes, 2002, 2005). In this context, the concept of euthymia might represent an important outcome for patients suffering from pain conditions. Euthymia includes affective and hedonic dimensions of subjective well-being (including restorative sleep), and psychological well-being, which entails an integration and balance of psychic forces (i.e., flexibility), a unifying outlook on life that guides behaviours and feelings to fashion the future consistently (i.e., consistency), resilience and tolerance to frustration and anxiety (i.e., resistance to stress; Carrozzino et al., 2021; Kusier and Folker, 2020; Fava and Bech, 2016; Fava and Guidi, 2020; Guidi and Fava, 2022).

A paucity of studies investigated well-being domains in patients with inflammatory rheumatic diseases, reflecting mainstream research focusing on negative functioning and likely glossing over the unique contributions of positive functioning (Wood and Tarrier, 2010). Nonetheless, there is evidence showing that over half of people with arthritis report high levels of emotional, psychological, and social well-being (Almweisheer et al., 2023) even those living with disabling chronic pain (Fuller-Thomson et al., 2023), and to a lesser extent (38%) in those with recurrent pain (Trompetter et al., 2019). To the best of our knowledge, euthymia has never been examined in people with inflammatory rheumatic disease, but research on other chronic health conditions suggests that lower levels of euthymia are associated with worse clinical conditions (Carrozzino et al., 2019; Cosci et al., 2021; Guidi et al., 2019; Zhang et al., 2022). Moreover, euthymia is positively associated with several dimensions of psychological well-being, suggesting only a partial overlap between the two constructs (Carrozzino et al., 2019).

A research tradition that emphasizes the processes by which people can live a fulfilling life despite pain is the one focusing on psychological flexibility (Goubert and Trompetter, 2017). Psychological flexibility allows individuals to accept inner experiences, including negative ones, while remaining sensitive to their direct experiences and engaging in values-based actions consistent with personal values (McCracken and Morley, 2014). Conversely, psychological inflexibility refers to a “rigid dominance of psychological reactions over chosen values and contingencies in guiding actions” (Bond et al., 2011, p. 678). Thus, it is strictly related to experiential avoidance, as individuals unwilling to stay in contact with unpleasant inner experiences are likely to take action to alter or avoid them (Bond et al., 2011; Hayes et al., 1996). The psychological (in)flexibility model posits that psychological inflexibility (e.g., experiential avoidance cognitive fusion, pain acceptance) is related to decreased engagement in values-based action, leading to reduced well-being (Hayes et al., 2012). In chronic pain patients, experiential avoidance predicted several outcomes, including depression and pain-related anxiety, over and beyond pain intensity, pain acceptance, and mindfulness (McCracken and Zhao-O’Brien, 2010). Although the psychological flexibility model has been successfully extended to chronic pain patients (McCracken and Vowles, 2014; Vowles et al., 2007, 2014), few studies investigated whether psychological (in)flexibility might explain associations between pain and psychological or physical outcomes. In chronic pain patients, the association between pain and psychological distress was mediated by cognitive fusion (Carvalho et al., 2019) and experiential avoidance (Goldbart et al., 2021). In patients with fibromyalgia and obesity, the pain severity-disability association was accounted for by pain acceptance (Varallo et al., 2022). The role of psychological inflexibility was also investigated in cancer patients, where cancer-related pain was positively related to psychological distress through cognitive fusion, experiential avoidance, and functional impairment (Brown et al., 2020). Overall, this initial evidence suggests that psychological flexibility model may successfully explain the relationship between pain and psychological distress.

Since pain is a complex phenomenon implying both physical and affective dimensions (Lumley et al., 2011), adaptive or dysfunctional responses to pain may rely on emotion regulation strategies used by people suffering from pain conditions. According to the process model, cognitive reappraisal and expressive suppression are the most used emotion regulation strategies (Gross, 2008, 2015). There is evidence showing that cognitive reappraisal and expressive suppression were not directly associated with pain, but expressive suppression was associated with higher anxiety and depression (Koechlin et al., 2018), and cognitive reappraisal predicted psychological distress (Karademas et al., 2020). Previous research suggested the importance of cognitive reappraisal in modulating the emotional component of episodic pain in rheumatoid arthritis (Hamilton et al., 2005, 2007). Specifically, emotion regulation and affective intensity moderated the prospective associations between pain and both positive and negative affect, suggesting that patients could recover from arthritic pain, except for those with difficulties in regulating strong unpleasant emotions (Hamilton et al., 2005). Thus, intense unpleasant emotions may not necessarily lead to emotion dysregulation in patients with good emotion regulation abilities (Hamilton et al., 2007). A transdiagnostic perspective on psychological inflexibility and emotion regulation has been recently proposed (Faustino, 2021). Faustino (2021) found that cognitive fusion was negatively associated with cognitive reappraisal both in the clinical and non-clinical sample, whereas it was positively associated with emotion suppression only in the non-clinical sample. Moreover, cognitive reappraisal predicted cognitive fusion in both samples, suggesting that individuals who lack cognitive reappraisal abilities are more likely to be fused with their inner experience (Faustino, 2021). Indeed, cognitive reappraisal reflects a shift from an evaluation to another one (i.e., reappraisal); to do that, “individuals must have the ability to distance themselves from the first evaluation. It is the ability to shift internal dispositions accordingly with context-dependent demands that underlies psychological flexibility” (Faustino, 2021, p. 10).

The overall objective of the present study was to investigate how pain intensity may be associated with well-being and why some patients, but not others, may live well despite arthritis-related pain. Particularly, we focused on pain related to three autoimmune inflammatory rheumatic diseases, namely rheumatoid arthritis, psoriatic arthritis, and axial spondyloarthritis. These diseases share several characteristics, including pain, stiffness, fatigue, decreased physical function, and potential deformities and joint destruction (Mease et al., 2019). Previous research on the mediation role of psychological flexibility solely focused on outcomes related to negative functioning (Carvalho et al., 2019; Goldbart et al., 2021; Varallo et al., 2022). The current study sought to extend current knowledge by considering euthymia as a relevant outcome. Specifically, we hypothesized that the unwillingness to stay in contact with unpleasant inner experiences and the tendency to take action to alter or avoid them (i.e., experiential avoidance) may explain the association between pain intensity and euthymia. Drawing upon the transdiagnostic perspective of emotion regulation and previous research (Faustino, 2021; Hamilton et al., 2005, 2007), we further hypothesized that cognitive reappraisal might represent a protective factor by moderating the association between pain intensity and euthymia through experiential avoidance. Thus, the following statistical hypothesis was derived: pain intensity would be positively associated with experiential avoidance (path *a*), which in turn would be associated with lower levels of euthymia (path *b*). Cognitive reappraisal would moderate the indirect effect, such that at higher levels of cognitive reappraisal, the association between pain and experiential avoidance would be weaker, which in turn would be associated with higher euthymia.

## 2 Methods

### 2.1 Participants and procedure

This is a cross-sectional study with patients recruited consecutively at the Immuno-rheumatology Unit, Campus Bio-Medico University of Rome. Patients were asked to participate in the study if they met the following inclusion criteria: (a) diagnosis of either rheumatoid arthritis, psoriatic arthritis, or axial spondyloarthritis/ankylosing spondylitis confirmed through ACR-EULAR, CASPAR, and ASAS classification criteria, respectively; (b) ability to understand and speak Italian; (c) aged 18 or older; (d) reading and signing informed consent. Exclusion criteria included: (a) current or past diagnosis of psychiatric disorder; (b) current or recent (within 1 year) diagnosis of cancer; (c) current infective disease; (d) currently in psychotherapy; (e) past psychotherapy for at least 6 months within the last 6 years.

The participants were recruited during routine follow-up visits, which were conducted by rheumatologists twice a week, from May 2020 to November 2021. Before each follow-up visit, rheumatologists verified whether eligibility criteria were met by reviewing clinical records. For patients who met eligibility criteria, a research assistant presented the study and provided the informed consent after the follow-up visit. The points of the informed consent were verbally explained, including anonymity and the right to decline to participate or withdraw from the study. All invited patients agreed to participate. The study was approved by the ethics committee of Campus Bio-Medico University of Rome (n. 77/19 OSS), complied with the Declaration of Helsinki, and adhered to Strengthening the Reporting of Observational Studies in Epidemiology (STROBE) guidelines for cross-sectional studies (Supplementary Table S1) (Vandenbroucke et al., 2007).

### 2.2 Measures

Clinical data (i.e., time since diagnosis, type of disease, number of comorbidities, presence/absence of fibromyalgia) were extracted from patients’ clinical records. Sociodemographic characteristics (i.e., age, gender, marital status, education level) and psychological measures were self-reported.

#### 2.2.1 Euthymia

The Euthymia Scale (Carrozzino et al., 2019, 2021) is composed by 10 items, which are scored as a False/True response format. Sample items include “I am able to adjust to changing situations” and “I generally feel cheerful and in good spirits.” Higher scores indicate higher levels of euthymia.

#### 2.2.2 Experiential avoidance

The Acceptance and Action Questionnaire-II (AAQ-II) is a 7-item self-report questionnaire designed to measure experiential avoidance (Bond et al., 2011; Pennato et al., 2013). Each item is rated on 7-point Likert scale (1 = never true; 7 = always true). Sample items include “I worry about not being able to control my worries and feelings” and “My painful experiences and memories make it difficult for me to live a life that I would value.” Higher scores indicate a greater level of experiential avoidance. Internal consistency in the original sample was good (Cronbach’s alpha = 0.88; Bond et al., 2011), while it was excellent in the present sample (Cronbach’s alpha = 0.93).

#### 2.2.3 Cognitive reappraisal

The Emotion Regulation Questionnaire was designed to measure individual differences in the usual adoption of cognitive reappraisal and expressive suppression (Balzarotti et al., 2010; Gross and John, 2003). For the purpose of this study, only the cognitive reappraisal subscale was used. It is composed of 6 items measured on a 7-point likert scale (1 = strongly disagree; 7 = strongly agree). Sample items include “When I want to feel less negative emotion, I change the way I’m thinking about the situation” and “When I’m faced with a stressful situation, I make myself think about it in a way that helps me stay calm.” In the original validation samples, Cronbach’s alpha ranged from 0.75 to 0.82 (Gross and John, 2003). In the present study, Cronbach’s alpha was 0.83.

#### 2.2.4 Pain intensity

Intensity of arthritic pain was measured with a Numeric Rating Scale (NRS) ranging from 0 (“no pain”) to 10 (“pain as bad as possible”). The patients were asked to rate their pain intensity in the last week. This instrument is commonly used to assess pain in arthritis (Hawker et al., 2011).

### 2.3 Data analysis

All statistical analyses were performed using IBM SPSS for Windows (version 22). Occasional missing values were imputed by computing the mean score of the respective sub-scale for each participant. Significant missing values were treated with listwise deletion (*N* = 26). Thus, analyses were conducted on 97 participants. A post-hoc power analysis (Cohen, 1988) with GPower 3.1.9.7 (Faul and Erdfelder, 1992) was conducted to check for adequacy of achieved power after excluding participants with significant missing values. Power was calculated as a function of population effect size (medium: *f^2^* = 0.15), significance level (*α* = 0.05), sample size (*N* = 97), and number of tested predictors (i.e., experiential avoidance, cognitive reappraisal, pain intensity, type of disease) resulting in a statistical power of 0.83.

Univariate outliers were identified through z scores greater than 3.29 (*p* < 0.001; Tabachnick et al., 2007), whereas multivariate outliers through Mahalanobis distance (*D*^2^), Cook’s distance, and leverage values. Criteria for multivariate outliers were: (a) cases with a *D*^2^ value greater than 20.515 (i.e., *D*^2^ value at *p* < 0.001, 5 degrees of freedom; Tabachnick et al., 2007); (b) Cook’s distance larger than 1.00 (Tabachnick et al., 2007); (c) leverage values greater than 3(*p* + 1)/*N* = 0.1856 (*p* = number of predictors; *N* = sample size; Howell, 2012).

Independence of observations was checked through Durbin-Watson statistic. Linearity was verified through a scatterplot of studentized residuals and unstandardized predicted value for independent and dependent variables collectively. Homoscedasticity was checked through inspection of the latter scatterplot and through Breusch-Pagan test. Normality of residuals was assessed by inspecting histogram and P–P plot (Clement and Bradley-Garcia, 2022), and by following recommendations of Kim (2013) for medium-sized samples (i.e., 50 < n < 300); specifically, normality assumption was met if the absolute z-values of skewness and kurtosis were smaller than 3.29. No outliers were identified and all assumptions were met.

Descriptive statistics were performed for all variables (i.e., sociodemographic, clinical, and psychological) by calculating means and percentages for continuous and categorical variables, respectively. Pearson correlations were computed.

Moderated mediation analysis was carried out by means of PROCESS macro (Model 7; Hayes, 2013). According to the study hypothesis, pain intensity was regarded as the independent variable, euthymia as the dependent variable, experiential avoidance as the mediator, and cognitive reappraisal as the moderator. Type of disease was dummy coded (0 = rheumatoid arthritis; 1 = psoriatic arthritis; 2 = axial spondyloarthritis) and was included as a covariate to control for differences among participants, with rheumatoid arthritis serving as the reference group. Interaction variables were mean centered (Aiken and West, 1991) and simple slope analysis was used to estimate the conditional indirect effect of pain intensity on euthymia through experiential avoidance at low (−1 SD), moderate (mean), and high (+1 SD) values of the moderator. The index of moderated mediation was used to test the moderation of the indirect effect (Hayes, 2015). All models were performed with a 5,000 bootstrap sample as recommended by Hayes (2013).

## 3 Results

### 3.1 Participants characteristics

A total of 123 patients agreed to participate and completed the questionnaires. Since 26 participants were excluded due to significant missing values (listwise deletion), the final sample comprised 97 participants. Demographic and clinical characteristics of the sample are shown in Table 1. Patients had an average age of 53. The majority were women, married or cohabitant, and completed high school. With regards to clinical characteristics, patients were diagnosed since 9.19 years and most of them did not have secondary fibromyalgia, although 61.9% had at least one comorbid medical condition.

**Table 1:** Demographic and clinical characteristics.

Age (years), *M* (*SD*)	53.37 (13.21)
Time since diagnosis (months), *M* (*SD*)	110.32 (79.55)
Gender, *N* (%)	
Women	73 (75.26)
Men	24 (24.74)
Marital status, *N* (%)	
Married/cohabitant	69 (71.13)
Unmarried	17 (17.53)
Separated/divorced	5 (5.15)
Widowed	5 (5.15)
Missing	1 (1.03)
Education level, *N* (%)	
Elementary school	7 (7.22)
Middle school	18 (18.56)
High school	46 (47.42)
Bachelor	19 (19.59)
Post-graduate education	7 (7.22)
Type of disease, *N* (%)	
Rheumatoid arthritis	38 (39.18)
Psoriatic arthritis	38 (39.18)
Spondyloarthritis	21 (21.65)
Fibromyalgia, *N* (%)	
Yes	24 (24.74)
No	71 (73.20)
Comorbidity (number of diseases), *N* (%)	
0	35 (36.08)
1	21 (21.65)
2	20 (20.62)
3	10 (10.31)
4	6 (6.19)
5	3 (3.09)
Missing	2 (2.06)

### 3.2 Correlation analyses

The correlation analyses showed that pain intensity was positively correlated with experiential avoidance (*r* = 0.39, *p* < 0.001) and negatively with euthymia (*r* = −0.35, *p* < 0.001). Experiential avoidance was negatively correlated with euthymia (*r* = −0.48, *p* < 0.001), whereas higher levels of cognitive reappraisal were significantly associated with higher levels of euthymia (*r* = 0.21, *p* < 0.05). The association of cognitive reappraisal with experiential avoidance was not significant. All correlations are detailed in Supplementary Table S2.

#### 3.2.1 Moderated mediation analysis

**Figure 1:**
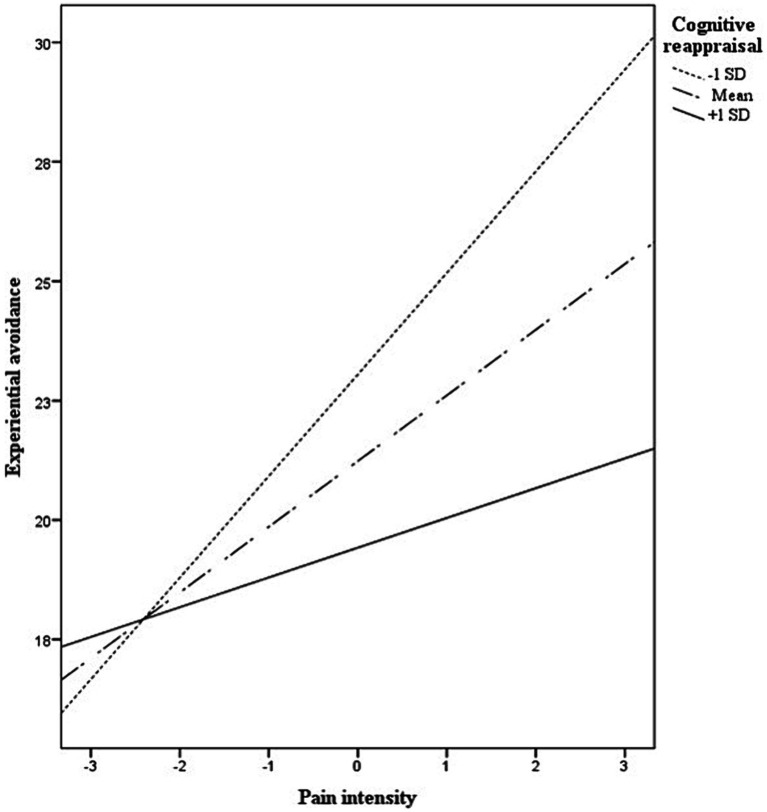
Moderation of cognitive reappraisal on the pain-experiential avoidance association.

**Figure 2:**
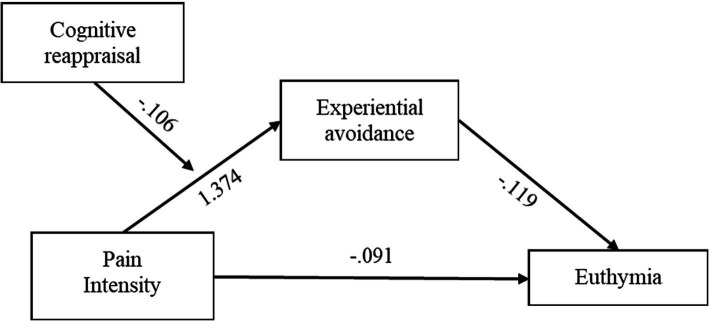
Conceptual moderated mediation model with coefficients of bootstrap results for regression model parameters.

The moderated mediation analysis is shown in Table 2. The explained variance of the overall model was 35%, *R*^2^ = 0.35, *F*(4,92) = 13.98, *p* < 0.001. Specifically, the direct effect of pain intensity on euthymia (path *c’*) was not significant after controlling for experiential avoidance, cognitive reappraisal, its interaction with pain intensity, and type of disease (*p* = −0.09). The conditional indirect effect of pain intensity on euthymia by way of experiential avoidance was significant in patients with low (i.e., one *SD* below the mean = −7.10) and medium (i.e., mean = 0) levels of cognitive reappraisal. This effect was not significant in those with high levels (i.e., above the mean = 7.10) of cognitive reappraisal. The index of moderated mediation was significant (Table 2). Overall, the higher the cognitive reappraisal, the lower the effect of pain on euthymia through experiential avoidance. A graphical representation of the association between pain and experiential avoidance (path *a*) at different levels of cognitive reappraisal is provided in Figure 1. The coefficients of bootstrap results for regression model parameters are shown in Figure 2. We further explored whether the moderated mediation model was affected by covariates such as age and time since diagnosis. This analysis showed that the model did not substantially change after controlling for these covariates, *R*^2^ = 0.39, *F*(6,87) = 9.98, *p* < 0.001; index of moderated mediation = 0.01, *CI_95%_* = [0.001, 0.024].

**Table 2:** Moderated mediation model.

	*B*	SE	*t*	95% Boot*CI*
Path *a*: Pain → Experiential avoidance	1.37***	0.35	3.90	[0.675, 2.074]
Interaction: Pain×Reappraisal → Experiential avoidance	−0.11*	0.05	−2.08	[−0.196, −0.016]
Path *b*: Experiential avoidance → Euthymia	−0.12***	0.02	−5.12	[−0.165, −0.073]
Path *c’* (direct effect): Pain → Euthymia	−0.09	0.08	−1.14	[−0.249, 0.068]
Conditional indirect effect: Pain → Experiential Avoidance → Euthymia Cognitive reappraisal:				
Low (−1 SD)	−0.25	0.07		[−0.399, −0.121]
Mean	−0.16	0.05		[−0.275, −0.074]
High (+1 SD)	−0.07	0.06		[−0.195, 0.033]
Index of moderated mediation	−0.01	0.01		[0.002, 0.024]

Covariates: Type of disease (rheumatoid arthritis = 0; psoriatic arthritis = 1; spondyloarthritis = 2).

*p < 0.05; **p < 0.01; ***p < 0.001.

## 4 Discussion

The main objective of this study was to investigate the link of pain intensity with well-being (i.e., euthymia) and to identify factors that contribute to better adaptation despite pain in patients with autoimmune inflammatory rheumatic diseases. Specifically, this study focused on the extent to which cognitive reappraisal, as an individual response of emotion regulation, moderates the association between pain and euthymia through experiential avoidance.

Previous research has identified psychological (in)flexibility-related constructs, such as cognitive fusion, experiential avoidance or pain acceptance, as mediators of the association between pain and disability in individuals with fibromyalgia (Varallo et al., 2022), as well as between pain and psychological distress in chronic pain (Carvalho et al., 2019; Goldbart et al., 2021) and cancer patients (Brown et al., 2020). The present study extends this knowledge beyond negative functioning by exploring how pain, cognitive reappraisal, and experiential avoidance contribute to positive outcomes, specifically euthymia, in patients with autoimmune inflammatory rheumatic diseases. Our findings indicate that patients with higher pain intensity reported increased experiential avoidance, which was associated with lower levels of euthymia. This suggests that an unwillingness to stay in contact with unpleasant inner experiences and taking action to alter or avoid them (Bond et al., 2011; Hayes et al., 1996) may explain the association between pain intensity and reduced euthymia. These results align with the psychological flexibility model (Hayes et al., 2012), which posits that psychological inflexibility is related to a lowered engagement in values-based action, thereby decreasing well-being (Hayes et al., 2012). Furthermore, the pain experience within this framework underscores how distressing cognitive content can dominate behaviour: “compelling cognitive content, such as this pain is terrible, my life is hopeless, or I am a complete failure, can have an overwhelming effect in experience where only behavior that follows or obeys what this content says can occur” (McCracken and Morley, 2014, p. 225).

Significantly, the negative effect of pain intensity on euthymia via experiential avoidance diminished as cognitive reappraisal increased. Thus, patients who effectively used cognitive reappraisal to regulate their emotions experienced improved well-being and a reduced negative impact of pain through decreased experiential avoidance. Notably, the indirect effect of pain intensity on euthymia became non-significant at high levels of cognitive reappraisal, suggesting that cognitive reappraisal may serve as a protective factor for patients with autoimmune inflammatory rheumatic diseases.

Cognitive reappraisal refers to a shift from an evaluation of an emotion-eliciting situation to another one, with a subsequent alteration of the emotional response (Faustino, 2021; Gross and John, 2003). Thus, it inherently reflects the ability of individuals in distancing from the first evaluation (Faustino, 2021). This process of distancing from initial evaluations and reappraising it in new ways may help patients manage painful stimuli and maintain higher levels of euthymia. Conversely, patients who do not engage in cognitive reappraisal may react to pain by avoiding unpleasant experiences, resulting in lower euthymia.

These findings have several clinical implications. Rather than solely targeting pain intensity, which often persists despite treatments (Carpenter et al., 2020; Sturgeon and Zautra, 2016; Sullivan and Ballantyne, 2016), improving euthymia may be achieved by addressing experiential avoidance and enhancing cognitive reappraisal. Acceptance and commitment therapy (ACT), which focuses on promoting behaviours congruent with one’s values despite internal unpleasant experiences (McCracken and Vowles, 2014), may be particularly effective. A recent systematic review showed that ACT was effective in improving emotional distress and physical functioning in patients with rheumatic diseases (Hegarty et al., 2020). However, the review included only patients with fibromyalgia and/or osteoarthritis, suggesting a lack of evidence for other rheumatic diseases. Future studies should consider extending these results in patients with rheumatoid arthritis, psoriatic arthritis, and axial spondylarthritis.

Cognitive reappraisal may be a valuable protective factor that may prevent an escalation from pain intensity to experiential avoidance, thus supporting higher euthymia. This is particularly significant considering that emotional dysregulation and past traumatic experiences typically co-occur in patients with chronic pain conditions (e.g., Nishimi et al., 2024), and are associated with increased somatic complaints (e.g., pain; Garnefski et al., 2017). Specific training fostering cognitive reappraisal may be helpful for patients with autoimmune inflammatory rheumatic diseases. Two tactics of reappraisal are distancing, involving a psychological distance from one’s construal of an emotional event, and reinterpretation, referring to the change of the meaning depicted in a stimulus (McRae et al., 2012). A longitudinal study found that both techniques reduced negative affect in healthy individuals, but only distancing showed a longitudinal reduction in perceived stress (Denny and Ochsner, 2014). Future studies may explore which cognitive reappraisal tactics may be most beneficial for patients with inflammatory rheumatic diseases to develop targeted interventions.

The results of this study should be interpreted in light of several limitations. First, the cross-sectional design of this study does not allow causal inferences. Future studies with a longitudinal design should investigate the directionality of associations between variables. Nonetheless, it is worth noting that proposed models are theoretically grounded. Second, this study relied solely on self-report measures and did not include other psychological or pain-related variables (e.g., nociceptive, neuropathic, nociplastic) that might be associated with euthymia. Third, the proposed models included only the type of autoimmune inflammatory rheumatic disease as a covariate; however, we also explored age and time since diagnosis as additional covariates. The analysis involving these additional covariates should be interpreted with caution due to limited statistical power. Future studies with a larger sample size should consider additional potential covariates to determine whether the proposed model remain significant. Fourth, a limitation is the reliance on a single measure of psychological inflexibility-related constructs. Future research could provide more comprehensive insights into the proposed associations by investigating multiple dimensions of psychological (in)flexibility (Landi et al., 2021). Furthermore, the study sample was consecutively recruited from a single clinical center, which may limit the generalizability of the findings. Finally, it should be emphasized that the study’s sample comprised patients with a variety of autoimmune inflammatory rheumatic diseases. Although the type of disease was included as a covariate in the statistical models, the associations between variables may still differ across specific diagnoses (i.e., rheumatoid arthritis, psoriatic arthritis, axial spondyloarthritis). Future research should further investigate these associations within more homogeneous subgroups to better understand how different inflammatory rheumatic disease might influence the associations among study variables. Despite these limitations, the study provides valuable and theoretically grounded insights into the role of cognitive reappraisal and experiential avoidance in managing pain in patients with autoimmune inflammatory rheumatic diseases. We found that higher pain intensity was associated with lower well-being (i.e., euthymia) through increased experiential avoidance. However, cognitive reappraisal moderated this effect, reducing the negative impact of pain. These findings highlight the potential of interventions that target experiential avoidance and enhance cognitive reappraisal to improve well-being. Future research should refine these approaches and examine their effectiveness across different rheumatic conditions.

## Data Availability

The raw data supporting the conclusions of this article will be made available by the authors, without undue reservation.
