# Supplementary material for: Euthymic despite pain: the role of cognitive reappraisal and experiential avoidance in autoimmune inflammatory rheumatic diseases—a cross-sectional study
Source: Front Psychol. 2024 Oct 4;15:1467555. doi: 10.3389/fpsyg.2024.1467555 (PMC11486703; doi:10.3389/fpsyg.2024.1467555)
Supplement: Supplementary file 2 [file Table_2.docx]

| **Table S2.** *Pearson correlations between study variables* | | | | |
| --- | --- | --- | --- | --- |
|  | 1. | 2. | 3. | 4. |
| 1. Pain Intensity | 1 |  |  |  |
| 2. Cognitive Reappraisal | -.01 | 1 |  |  |
| 3. Experiential avoidance | .39^***^ | -.16 | 1 |  |
| 4. Euthymia | -.35^***^ | .21^*^ | -.48^***^ | 1 |
| *Note*. * *p* < .05; ** *p* < .01; *** *p* < .001. | | | | |
